# Massively multiplayer online role-playing games: comparing characteristics of addict *vs *non-addict online recruited gamers in a French adult population

**DOI:** 10.1186/1471-244X-11-144

**Published:** 2011-08-26

**Authors:** Sophia Achab, Magali Nicolier, Frédéric Mauny, Julie Monnin, Benoit Trojak, Pierre Vandel, Daniel Sechter, Philip Gorwood, Emmanuel Haffen

**Affiliations:** 1 Clinical Psychiatry Department, Besançon University Hospital, 25030 Besançon Cedex, France; 2 EA 481 "Neurosciences Laboratory"- Franche-Comté University, 1 place du maréchal Leclerc, 25030 Besançon Cedex, France; 3 Clinical Psychiatry Department, Addictological Unit of Geneva University Hospital, 70C rue du Grand-Pré Geneva, Switzerland; 4 Medical Information Department, Besançon University Hospital, 25030 Besançon Cedex, France; 5 UMR CNRS 6249 « Chrono Environnement » -Franche-Comté University, 16 route de Gray, 25030 Besançon Cedex, France; 6 INSERM Technological Innovation Clinical Investigation Center (INSERM CIC-IT 808), Besançon University Hospital, 25030 Besançon Cedex, France; 7 Clinical Psychiatry Department, Dijon University Hospital, 1 Bd Jeanne d'Arc, BP 77908 21079 Dijon Cedex, France; 8 Saint-Anne Hospital (Paris Descartes), 100 rue de la santé, 75674 Paris Cedex 14, France; 9 INSERM U894, Paris Descartes University, Paul Broca Centre, 2 ter rue d'Alésia, 75014 Paris, France

## Abstract

**Background:**

Massively Multiplayer Online Role-Playing Games (MMORPGs) are a very popular and enjoyable leisure activity, and there is a lack of international validated instruments to assess excessive gaming. With the growing number of gamers worldwide, adverse effects (isolation, hospitalizations, excessive use, etc.) are observed in a minority of gamers, which is a concern for society and for the scientific community. In the present study, we focused on screening gamers at potential risk of MMORPG addiction.

**Methods:**

In this exploratory study, we focused on characteristics, online habits and problematic overuse in adult MMORPG gamers. In addition to socio-demographical data and gamer behavioral patterns, 3 different instruments for screening addiction were used in French MMORPG gamers recruited online over 10 consecutive months: the substance dependence criteria for the Diagnostic and Statistical Manual of Mental Disorder, fourth revised edition (DSM-IV-TR) that has been adapted for MMORPG (DAS), the qualitative Goldberg Internet Addiction Disorder scale (GIAD) and the quantitative Orman Internet Stress Scale (ISS). For all scales, a score above a specific threshold defined positivity.

**Results:**

The 448 participating adult gamers were mainly young adult university graduates living alone in urban areas. Participants showed high rates of both Internet addiction (44.2% for GIAD, 32.6% for ISS) and DAS positivity (27.5%). Compared to the DAS negative group, DAS positive gamers reported significantly higher rates of tolerance phenomenon (increased amount of time in online gaming to obtain the desired effect) and declared significantly more social, financial (OR: 4.85), marital (OR: 4.61), family (OR: 4.69) and/or professional difficulties (OR: 4.42) since they started online gaming. Furthermore, these gamers self-reported significantly higher rates (3 times more) of irritability, daytime sleepiness, sleep deprivation due to play, low mood and emotional changes since online gaming onset.

**Conclusions:**

The DAS appeared to be a good first-line instrument to screen MMORPG addiction in online gamers. This study found high MMORPG addiction rates, and self-reported adverse symptoms in important aspects of life, including mood and sleep. This confirms the need to set up relevant prevention programs against online game overuse.

## Background

Dependence involves a complex system of bio psychosocial factors affecting individuals, their actions and their culture, and has also been referred to as a syndrome with multiple expressions [1]. In 1964 the World Health Organization (WHO) introduced the concept of dependence to replace addiction and habituation. The term dependence can be used generally with reference to the whole range of psychoactive drugs (drug, chemical or substance use dependence) or with specific reference to a particular drug or class of drugs (alcohol or opioid dependence) and refers to both physical and psychological elements [2]. According to the Diagnostic and Statistical Manual of Mental Disorders, Fourth Edition-Text Revision (DSM-IV-TR), substance dependence may involve several symptoms (tolerance, withdrawal, adverse repercussions on social and professional areas, loss of control of the consumption, and persistence despite the adverse effects engendered). The concept has since undergone major revision with the appearance of new types of entities, entitled non-chemical (*i.e*. behavioral) addictions such as eating disorders, compulsive buying, exercise abuse [3] and pathological gambling. Clinicians tend to distinguish between abuse, dependence and addiction, referring to either substance or behavior. This distinction is sustained by recent neurobiological findings on the different neuronal processes involved in dependence or addiction [4]. Dependence could be defined as an adaptive neural response to the pharmacological effect of substance abuse, and is associated with withdrawal when the substance is not accessible. This definition corresponds to what was previously called "physical dependence", that is inadequate to explain substance and non-substance addiction. The previous "psychological dependence" is more likely to be a "choice disorder" in the addiction disorder, in which loss of control and inadequate decision making leads to an automatic and compulsive behavior which is pursued despite adverse psychological, physical and/or social consequences [5]. Several types of behavior, besides psychoactive substance use, produce a short-term reward that may engender persistent behavior, despite knowledge of adverse consequences. These disorders have been conceptualized as lying along an impulsive-compulsive spectrum or an addiction spectrum such as "behavioral" addictions. In support of the second hypothesis, growing evidence suggests that behavioral addictions resemble substance addictions in many domains, including natural history, phenomenology, tolerance, and comorbidity, overlapping genetic contribution, neurobiological mechanism, and response to treatment [6]. The American Psychiatric Association (A.P.A) press release quoted O'Brien, chair of the Substance-Related Disorders Work Group, as saying: "substance research supported that pathological gambling and substance use disorders were very similar because both were related to poor impulse control and brain's system of reward and aggression" [7]. These findings support the forthcoming DSM fifth edition (DSM-V) that may propose a new category of Addiction and Related Disorders encompassing both use disorders and non-substance addictions. Current data suggest that this combined category may be appropriate for pathological gambling and a few well studied behavioral addictions, *e.g*. Internet addiction (IA) and video/computer game addiction [6]. IA was described as a joke by Goldberg in 1994 by reproducing the DSM-IV criteria for substance dependence [8]. Since then, this "new disorder" has been the subject of scientific interest until the recent call for it to join the ranks of DSM V classification. Davis's theoretical model on Problematic Internet Use (PIU) distinguishes two different entities: i) Specific PIU, related to a particular content and which could exist independently from the Internet vector, such as gambling and videogames and ii) Generalized PIU which is related to specific Internet content such as chats, e-mails and social networks [9]. The Internet has provided a wide range of possibilities for traditional video games and the attraction is clearly common worldwide, particularly with the Massively Multiplayer Online Role-Playing Games (MMORPGs). An example of this popularity is World of Warcraft^© ^(WoW), which has over 11.5 million active subscribers [10] and accounts for an estimated 62% of the online video game market [11]. These games are the latest Internet-only gaming experience, and are typically represented by large, sophisticated and evolving virtual worlds set in different environments [12]. However, as the popularity of MMORPG has grown, questions are being raised about their potential for excessive use. MMORPGs are played for much longer periods of time than other games [13], which could also indicate a potential for greater negative effects on players [14].

Up to now, little research has been carried out on online video games, particularly on the different aspects of online gaming, the characteristics of adult gamers and their addiction level. Previous studies mainly focused on the demographics of MMORPG gamers. Few studies have looked at the effects of MMORPG. Kim evaluated online game addiction using a modified version of Young's Internet Addiction Scale and observed a positive correlation between online game addiction and aggression and narcissistic personality traits, and a negative correlation between online game addiction and self-control [15]. In an exploratory study, Hussain examined the gender swapping phenomenon [16] and in a qualitative analysis using 71 online interviews, showed how gamers used MMORPG to alleviate negative feelings, providing detailed descriptions of personal problems that arise due to playing MMORPG in a third of subjects [17]. In another exploratory study, Longman separated 206 international participants into 2 groups according to time spent playing MMORPG per week, and observed that the high use group presented low levels of offline social support and high levels of negative psychological symptoms [18]. Another approach focused on the relationship between addiction and avatar, the game character [14]. In this international study, 15.4% of gamers were female and nearly 40% of 548 MMORPG players considered themselves addicted. Moreover, Mentzoni *et al*. observed that problematic use of video games was associated with lower scores for life satisfaction and high levels of anxiety and depression [19].

Video games are a highly attractive leisure activity, and can even be used in medical applications (pain, muscular rehabilitation, cognitive stimulation, etc.) [20], but they can also cause adverse effects such as addiction. To address this growing phenomenon, the government of South Korea, a pioneering country for MMORPG development, has just decided to introduce a midnight ban for young gamers with a lockout of 6 hours. In addition, their Internet connection would be slowed *via *spyware for players gaming for more than 6 hours (http://www.mmorpg.com/newsroom.cfm/read/16704). In the same way and with an estimated 10 million Internet-addicted teenagers, China has begun restricting computer game use: current laws discourage more than 3 hours of daily game use [21]. MMORPG addiction is an emerging phenomenon (which has been acknowledged for a decade) in a context of controversy surrounding theories on behavioral addictions and "conceptual chaos" in the field of addictions [22]. Currently, no scientifically established and unanimously recognized classification for diagnosing online video game addiction exists [23].

In the absence of gold standard diagnostic criteria for IA and online gaming addiction, we referred to DSM-IV-TR criteria for substance dependence. Many studies in these fields have adapted criteria from DSM-IV-TR such as gambling criteria for the Internet Addiction Test (IAT) [24], substance dependence and gambling criteria for Problem Videogame Playing (PVP) [25]. Emerging data point to clinical and neurobiological similarities between substance use disorders and behavioral addictions [26,27]. We decided to test substance dependence DSM-IV-TR criteria to screen for online gaming addiction.

In this context, this research, which is mostly exploratory, focused on separating MMORPG addiction from IA (according to Davis's theoretical model) using different screening tools for addiction in the same sample. To address MMORPG addiction, we used the DSM-IV-TR criteria for substance dependence [28,29] which we adapted for online video gaming (replacing the term "substance" by the term "online video games"). To address IA, 2 different scales were used: the qualitative Goldberg Internet Addiction Disorder (GIAD) [8] (including tolerance and withdrawal dependence criteria) and the quantitative Orman Internet Stress Scale (ISS) [30] (excluding tolerance and withdrawal dependence criteria and focusing on addiction characteristics such as loss of control and adverse consequences of excessive Internet use). This study also focused on adult MMORPG gamers using an online recruitment design.

## Methods

### Study design

The target population of our study consisted of French MMORPG gamers aged over 18 and recruited online in discussion forum guilds often visited by the gamers. These forum guilds are entities created by groups of gamers seeking the same objectives in the most popular game, World of Warcraft (WoW). The self-administered nature of the questionnaire is, however, less robust than directed interviewing. Self-administered online questionnaires have been used in other studies in these fields and have been described as a satisfactory method [31].

The study protocol was approved by the Ethical Committee of Besançon University Hospital (authorization given by the General Health Administration: DGS2007-0382). To ensure anonymity, we sent an invitation summarizing our study, with a link to the personalized study website, to 234 guilds of WoW games between May 2009 and March 2010. Once gamers connected to the website, they had access to information on the researchers, aims of the study and clear instructions on the questionnaire, confidentiality and their right to withdraw at any time from the study. Questionnaires were strictly anonymous and confidential, and no data that could identify gamers was collected [*e.g*. Internet Protocol (IP) address which is a numerical label assigned to each computer participating in a computer network] according to French ethical standards. All subjects were volunteers and declared that they were aged 18 years or older. All responders consented to online study participation and authorized the researchers to use their incomplete data when necessary. The online questionnaire took 45 minutes to complete. The first part of the questionnaire, consisted of a 63-item self-administered list of questions assessing social and demographical data, the relationship between gaming and health, gaming and socio-professional consequences, and clinical criteria screening for IA and online game addiction. The questionnaire had a consistent pattern and participants had to answer each question to gain access to the following one. Participants answered yes or no to all questions except for the following: i) for the question "What are your qualifications?" gamers made a simple choice between 3 possibilities: below High School Diploma, between the High School Diploma and University degree and finally Master's degree or higher: ii) for the question "What are you looking for in MMORPG?" gamers made a choice between 6 possibilities that we subsequently grouped into 4 categories according to Bartle's taxonomy (1996) [32]: "*Explorer*" for "discovery" or "exploration" of the game environment, "*Achiever*" for "challenge" or "having a powerful avatar", "*Role player*" for "role playing in an alternative world", and "*Socializer*" for "interaction with other gamers"; iii) for the question "How does playing make you feel?" gamers made a choice between 3 possibilities: "greater personal satisfaction", "sense of power" and/or "sense of belonging to a group"; iv) for the question "Since you started gaming, do you feel" gamers made a simple choice between 5 possibilities: happier, more irritable, more anxious, less calm or more sad; v) for the question "At what age did you start playing?" gamers gave an open ended answer and vi) for the question "What sort of effects do you feel gaming is having on your health?" participants gave an open ended answer that we subsequently reclassified into 5 categories (i) no effect, ii) physical effects such as visual or musculoskeletal disorders, iii) psychological effects such as nervousness, iv) fatigue or insomnia and v) both physical and psychological effects).

### Different scales

The first part of the online questionnaire comprised 63 items including three Internet and online gaming screening instruments. Each scale has its own independent items. i) To assess online gaming addiction, we adapted the DSM-IV-TR for substance dependence with the same cut-off point (3 or more criteria) as the original in favor of a self-quoted diagnosis of online video game addiction. We called this scale the DSM-IV-TR substance dependence Adapted Scale (DAS) in this paper. It has 7 items (as for the original scale) associated with online game use. To assess IA, ii) the Goldberg Internet Addiction Disorder (GIAD) scale is a qualitative scale with 11 items and was adapted from the DSM-IV substance dependence by Goldberg with the same cut-off point as DSM-IV substance dependence (3 or more criteria) indicating Internet addiction [8]. The withdrawal symptoms for this tool were "agitation", "continuous thoughts of the Internet" and "involuntary hand movements". iii) Orman's Internet Stress Scale (ISS) [30] is a quantitative scale with 9 items devoted to Internet addiction tendency. A score between 0 and 3 relates to a low tendency to addiction while a score between 4 and 9 corresponds to an addiction risk. The benefit of this tool was its IA severity screening, the absence of "tolerance" or "withdrawal" symptoms, the focus on the adverse consequences of Internet abuse, the self-reported unsatisfying Internet abuse and loss of control. This tool was more effective in screening for addiction than dependence.

For all items of the 3 scales, participants answered yes or no. For each scale, the subjects whose score reached the cut-off point were considered to be positive.

### Statistical analysis

A univariate analysis was performed using the two-sample t-test (continuous variables) and Pearson's chi-square test (unmatched categorical variables). To assess independent factors associated with the DAS score (recoded as positive or negative), a multivariate analysis was conducted using a logistic regression model. Age, sex and educational level were considered as potential confounding factors [14,15,17,33,34] and were systematically introduced in the logistic model (adjusted results). Independent factors associated with the DAS score on univariate analysis at p < 0.2 were separately introduced in the logistic model. The Benjamini and Hochberg procedure was used to control the effect of multiple comparisons [35,36]. The correction was applied to groups of simultaneous tests of null hypotheses. Analyses were considered as simultaneous when the independent variables described a characteristic from the same family (addiction scales, baseline demographics, social impairment, etc.). Corrected p-values and corrected Confidence Intervals were calculated in order to control the False Discovery Rate. The significance threshold was set to 0.05. Analyses were performed using SYSTAT software (v 12).

## Results

### Participants

Of the 861 visitors to the online website dedicated to this project, 516 completed the online questionnaire (59.9%). Sixty three records were excluded: 56 subjects had indicated that they did not agree to the data being used if their data were incomplete. More than 10% of the data were missing for 5 participants and 2 questionnaires presented inconsistent data when we compared several demographic characteristics (age/number of children/family status and age/educational level). Four hundred and forty eight responders were therefore included in this data analysis (52.6%).

### Characteristics of MMORPG players

Online game playing participant characteristics (n = 448) are listed in Table 1. According to the DAS Scale, 27.5% of subjects screened had positive addiction criteria to MMORPG (that we named DAS**^+^**) [95% Confidence Interval (95%CI): 23.3-31.6] (Table 2). With ISS and GIAD scales that measure IA, the positive groups (that we named ISS^+ ^and GIAD^+^) reached 32.6% [95%CI: 28.2-36.9] and 44.2% [95%CI: 39.6-48.8] respectively. We tested the association between DAS and IA scales (n = 448) (Table 2). DAS was statistically associated with GIAD and ISS (both with p corrected < 10^-3^): Although 77.5% of responses (n = 84 ISS^+^/DAS^+^, and n = 263 ISS^-^/DAS^-^) were concordant between DAS and ISS, and 72.5% between DAS and GIAD (n = 99 GIAD^+^/DAS^+ ^and n = 226 GIAD^-^/DAS^-^), divergences were also observed: 42% of ISS^+ ^were DAS^- ^and 50% of GIAD^+ ^gamers were DAS^-^.

**Table 1:** Baseline demographic characteristics of a sample of French Massively Multiplayer Online Role-Playing (MMORPG) Gamers

*Variable*	*Mean*	*S.D*.
**Age **(year)	26.6(18-54)	7.1
	***Number***	***%***
**Gender**		
Male	374	82.7
Female	78	17.3
**Marital status**		
Single	253	56.1
In a relationship	198	43.9
**Living alone**	353	78.2
**Children**	93	21
**Residence**		
Rural	93	20.7
Urban	357	79.3
**Geographical localization**		
Ile-de-France region	112	26
Rhone-Alpes region	48	11.2
Other	271	62.8
**Educational level**		
Below High School Diploma	43	9.6
High School Diploma to University degree	298	66.7
Master's degree and higher	106	23.7
**Occupation**		
None	27	6.2
Student	148	34.5
Worker	255	59.3
**Gamer type**		
Socializer	351	77.5
Achiever	317	70
Explorer	295	65.1
Role Player	140	30.9

**Table 2:** MMORPG gamers' comparison with (*vs *without) addiction according to the 3 screening addiction scales (n = 448)

			ISS threshold score		GIAD threshold score		N
			***over (+)***	***below (-)***	***over (+)***	***below (-)***	
			**n**	**n**	**n**	**n**	

**DAS threshold score**	*over (+)*	n	84	39	99	24	123
	*below (-)*	n	62	263	99	226	325
		n	146	302	198	250	448
**statistical test**		χ^2^	98.4		90.5		
		Corrected p value	< 10-3		< 10-3		

Based on the high concordance rate between IA screening scales and the MMORPG addiction screening scale, we present here only the results according to DAS.

### Distribution among DSM-IV TR addiction criteria

No significant difference was found between gamers from the positive group (over threshold: DAS**^+^**) in terms of age (25.7 years old, ranging from 18 to 46) and those from the negative group (below threshold: DAS**^-^**, 27 years old, ranging from 18 to 54); or in terms of gender (28.8% *vs *20.8% of women) (Table 3). DAS^+ ^gamers were less likely to be University graduates than DAS^- ^(Table 3).

**Table 3:** Baseline demographics of French MMORPG gamers and their DAS responses

Variables	Total sample of population		Over threshold DAS (+)		Below threshold DAS (-)		Statistical test	
	Mean	**S.D**.	Mean	**S.D**.	Mean	**S.D**.	Statistical tests	Corrected p-value
**Age (years)**	26.6	7.1	25.7	6.5	27	7.3	t = 1.845	0.099
	Number	%	Number	%	Number	%		
**Gender**								
male	374	82.7	107	28.8	264	71.2	χ^2 ^= 2.08	0.149
female	78	17.3	16	20.8	61	79.2	1df	
**Educational level**								
BHSD Δ	43	9.6	16	37.2	27	62.8	χ^2 ^= 6.441	0.12
UD Δ	298	66.7	86	29.3	208	70.7	2df	
MD Δ	106	23.7	20	18.9	86	81.1		

Δ BHSD: Below High School Diploma; UD: High School Diploma to university; MD: Master's degree and higher.

With adjustment for age, sex and educational level [14,15,17,33,34], the DAS score was significantly associated with a large number of variables in the following dimensions: social life, Internet and online gaming, emotional changes and health impairment. Firstly, considering social life since online gaming onset (Table 4), DAS^+ ^gamers self reported significantly higher rates of a "lack of other leisure activities" (p < 10^-3^, OR:0.22, 95%CI:0.13-0.36), of "going out less" (p < 10^-3^, OR:4.79, 95%CI:3.05-7.53), of "seeing fewer friends" (p < 10^-3^, OR:5.78, 95%CI:3.58-9.32) and of experiencing marital (p < 10^-3^, OR:4.61, 95%CI:2.66-7.99), family (p < 10^-3^, OR:4.69, 95%CI:2.80-7.86), work (p < 10^-3^, OR:4.42, 95%CI:2.56-7.64) and/or financial (p < 10^-2^, OR:4.85, 95%CI:1.18-19.97) difficulties compared to DAS^- ^gamers. A significantly higher proportion (p < 10^-3^) of these DAS^+ ^gamers also reported depriving themselves of necessary purchases in order to play MMORPG (OR: 6.05, 95%CI: 2.48-14.74). In the same way, they increased the amount of time spent on the Internet to obtain satisfaction for at least 12 months (OR: 2.99, 95%CI: 1.84-4.87) (Table 5) compared with gamers in the DAS^- ^group.

**Table 4:** Social impairment and DAS responses

Variables	Total sample of population		Over threshold DAS (+)		Below threshold DAS (-)		Univariate analysis		Multivariate analysis	
	**Number**	**%**	**Number**	**%**	**Number**	**%**	**Statistical tests**	**Corrected p-value**	**Odds Ratio (95%CI)**	**Corrected** **p-value**

Other hobbies										
no	83	18.4	46	56.1	36	43.9	χ^2 ^= 41.77	< 10-3	0.22 (0.13-0.36)	< 10-3
yes	367	81.6	76	20.9	288	79.1	1df			
Going out less										
no	282	62.5	44	15.7	236	84.3	χ^2 ^= 52.34	< 10-3	4.79 (3.05-7.53)	< 10-3
yes	169	37.5	79	47.3	88	52.7	1df			
See fewer friends										
no	340	75.4	60	17.8	277	82.2	χ^2 ^= 62.14	< 10-3	5.78 (3.58-9.32)	< 10-3
yes	111	24.6	62	56.4	48	43.6	1df			
Marital difficulties										
no	369	82.7	82	22.4	284	77.6	χ^2 ^= 31.18	< 10-3	4.61 (2.66-7.99)	< 10-3
yes	77	17.3	41	53.9	35	46.1	1df			
Family difficulties										
no	359	79.6	73	20.5	284	79.5	χ^2 ^= 41.86	< 10-3	4.69 (2.80-7.86)	< 10-3
yes	92	20.4	49	54.4	41	45.6	1df			
Work difficulties										
no	379	83.8	81	21.6	294	78.4	χ^2 ^= 39.61	< 10-3	4.42 (2.56-7.64)	< 10-3
yes	73	16.2	42	57.5	3	42.5	1df			
Financial difficulties										
no	443	98	117	26.7	322	73.3	χ^2 ^= 7.09	10^-2^	4.85 (1.18-19.97)	0.03
yes	9	2	6	66.7	3	33.3	1df			
Deprivation of necessary purchases in order to play										
no	422	94.6	105	25.1	314	74.9	χ^2 ^= 19.79	< 10-3	6.05 (2.48-14.74)	< 10-3
yes	24	5.4	16	66.7	8	33.3	1df			

Odds Ratio and p-value were adjusted for age, sex and educational level.

**Table 5:** Connecting to Internet and gaming and DAS responses

Variables	Total sample of population		Over threshold DAS (+)		Below threshold DAS (-)		Univariate analysis		Multivariate analysis	
	Mean	**S.D**.	Mean	**S.D**.	Mean	**S.D**.	Statistical test	Corrected p-value	Odds Ratio (95% CI)	Corrected p-value
**Hours per week**										
Internet	43.3	23.7	50.3	24.6	40.7	22.9	t = 3.77	< 10-3	1.18	< 10-3
	(10-100)								(1.08-1.29)	
MMORPG	30.3	18.7	36.8	22	27.7	16.7	t = 4.16	< 10-3	1.28	< 10-3
	(5-100)								(1.14-1.44)	
	Number	%	Number	%	Number	%				
**Self-reported type of engagement in online gaming**										
Casual	206	46	39	19.2	165	80.8	χ^2 ^= 34.91	< 10-3		
Hard Core	214	47.8	64	30.3	147	69.7	2df		1.70	< 10-3
									(1.07-2.71)	
No life	28	6.2	20	71.4	8	28.6			9.14	
									(3.69-22.64)	
**Increase of time spent on the Internet to obtain satisfaction for at least 12 months**										
no	354	78.1	78	22.3	272	77.7	χ^2 ^= 21.47	< 10-3	2.99	< 10-3
yes	99	21.9	45	45.9	53	54.1	1df		(1.84-4.87)	

Odds Ratio and p-value were adjusted for age, sex and educational level.

Secondly, focusing on gamer characteristics (Table 5), the DAS^+ ^group spent significantly (p < 10^-3^) more time on the Internet or gaming than the DAS^- ^group (OR: 1.18, 95%CI: 1.08-1.29 and OR: 1.28, 95%CI: 1.14-1.44 respectively). The addiction rates according to DAS were proportionally (p < 10^-3^) linked with the self-reported graduation of the amount of gaming engagement compared to casual gamers (OR:1.70, 95%CI:1.07-2.71) for hardcore gamers and (OR:9.14, 95%CI: 3.69-22.64) for no life.

In terms of the relationship between emotional changes and gaming (Table 6), the DAS^+ ^group felt a significantly (p<10-3) greater in-game sense of power compared to the DAS^- ^group (OR: 3.21, 95%CI:1.62-6.36). The DAS^+ ^group sought significantly greater personal satisfaction (p = 0.008, OR: 1.78, 95%CI: 1.16-2.73) from games than the DAS^- ^group. In the same way, those who felt a sense of group belonging in-game were more likely to be in the DAS^+ ^group (p = 0.026, OR: 1.63, 95%CI: 1.06-2.50).

**Table 6:** Self-reported emotional changes and DAS responses

Variables	Total sample of population		Over threshold DAS (+)		Below threshold DAS (-)		Univariate analysis		Multivariate analysis	
	**Number**	**%**	**Number**	**%**	**Number**	**%**	**Statistical tests**	**Corrected p-value**	**Odds Ratio (95%CI)**	**Corrected p-value**

										
**More personal satisfaction**										
no	251	55.4	55	22.3	192	77.7	χ^2 ^= 7.44	0.015	1.78	0.008
yes	202	44.6	68	33.8	133	66.2	1df		(1.16-2.73)	
**Sense of power**										
no	413	91.2	101	24.8	307	75.2	χ^2 ^= 16.73	< 10-3	3.21	<0.001
yes	40	8.8	22	55	18	45	1df		(1.62-6.36)	
**Sense of belonging to a group**										
no	277	61.1	65	23.6	210	76.4	χ^2 ^= 5.21	0.02	1.63	0.026
yes	176	38.9	58	33.5	115	66.5	1df		(1.06-2.50)	

Odds ratio and p-value were adjusted for age, sex and educational level.

We also assessed factors associated with gaming (Table 7). The DAS^+ ^group slept significantly less than the DAS^- ^group (p = 0.004, OR: 0.78, 95%CI: 0.66-0.93) and the number of those who did not get restful sleep was significantly higher (p < 10^-3^, OR:0.23, 95%CI:0.14-0.38). Sleep deprivation due to play (OR: 2.83, 95%CI: 1.83-4.38) and diurnal sleepiness (OR: 3.10, 95%CI: 1.92-5.00) was significantly (p < 10^-3^) associated with high rates of DAS positivity. For the question "how do you feel since you started playing?", and compared to gamers who were happier since they had started playing, DAS^+ ^gamers declared significantly more often (p < 10^-3^) that they were more irritable (OR: 2.56, 95%CI: 1.19-5.48), less calm (OR for "more calm":0.39, 95%CI: 0.22-0. 69) or more sad (OR: 12.48, 95%CI: 2.64-59.06). Furthermore, these gamers declared significantly (p < 10^-3^) more often confusing real life with the virtual than the DAS^- ^group (OR: 5.01, 95%CI: 2.21-11.34). We also studied the effects of gaming on health (Table 7). DAS^+ ^gamers self-reported suffering significantly more often than DAS^- ^gamers (p < 10^-3^) from psychological (OR: 3.21, 95%CI: 1.86-5.56) or physical (OR: 3.23, 95%CI: 1.44-7.22) or both psychological and physical effects (OR: 14.09, 95% CI: 2.89-68.61) due to gaming.

**Table 7:** Gaming and self-reported health impairment and DAS responses

Variables	Total sample of population		Over threshold DAS (+)		Below threshold DAS (-)		Univariate analysis		Multivariate analysis	
	**Mean**	**S.D**.	**Mean**	**S.D**.	**Mean**	**S.D**.	**Statistical tests**	**Corrected p-value**	**Odds Ratio (95%CI)**	**Corrected p-value**

**Effect on sleep**										
Hours slept per night	7.1 (4-14)	1.3	6.8	1.4	7.2	1.3	t = 2.74	0.043	0.78	0.004
									(0.66-0.93)	
	Number	%	Number	%	Number	%				
Restful sleep										
no	86	19.1	46	53.5	40	46.5	χ^2 ^= 36.82	< 10-3	0.23	< 10-3
yes	364	80.9	76	21.1	285	78.9	1df		(0.14-0.38)	
Deprivation of sleep due to play										
no	285	63.2	55	19.4	228	80.6	χ^2 ^= 24.01	< 10-3	2.83	< 10-3
yes	166	36.8	67	40.9	97	59.1	1df		(1.83-4.38)	
Daytime sleepiness										
no	348	77.3	74	21.5	270	78.5	χ^2 ^= 27.712	< 10-3	3.10	< 10-3
yes	102	22.7	49	48	53	52	1df		(1.92-5.00)	
**Effect on mood**										
Happier	87	24.1	31	36.5	54	63.5	χ^2 ^= 58.82	< 10-3	1	< 10-3
More irritable	45	12.5	27	60	18	40	4df		2.56	
									(1.19-5.48)	
More anxious	8	2.2	4	50	4	50			1.69	
									(0.39-7.34)	
More sad	17	4.7	15	88.2	2	11.8			12.48	
									(2.64-59.06)	
More calm	204	56.5	38	18.8	164	81.2			0.39	
									(0.22-0.69)	
**Effect on health**										
None	333	74.7	67	20.2	264	79.8	χ^2 ^= 37.41	< 10-3	1	< 10-3
Psychological effect	73	16.4	32	45.1	39	54.9	3df		3.21	
									(1.86-5.56)	
Physical effect	29	6.5	14	48.3	15	51.7			3.23	
									(1.44-7.22)	
Both effects	11	2.5	8	72.7	3	27.3			14.09	
									(2.89-68.61)	
**Confusing real life vs fiction**										
no	422	93.8	104	24.9	314	75.1	χ^2 ^= 20.51	< 10-3	5.01	< 10-3
yes	28	6.2	18	64.3	10	35.7	1df		(2.21-11.34)	

Odds ratio and p-value were adjusted for age, sex and educational level.

Finally, concerning gamer opinions of guilds (Table 8), the DAS^+ ^group felt that their guilds required them to spend a certain amount of time gaming and exerted pressure on them (OR:2.55, 95%CI:1.63-3.99 and OR:5.19, 95%CI:2.09-12.91 respectively). Concerning the role of guilds, gamers who felt their guild imposed demands on their time reported higher rates of DAS positivity (3.7 times more), and gamers who felt their guilds exerted pressure reported DAS positivity rates 2.6 times higher than gamers who did not feel this (data not shown).

**Table 8:** Effects of guilds and DAS responses

Variables	Total sample of population		Over threshold DAS (+)		Below threshold DAS (-)		Univariate analysis		Multivariate analysis	
**Effect of guilds**	**Number**	**%**	**Number**	**%**	**Number**	**%**	**Statistical tests**	**Corrected p-value**	**Odds Ratio (95%CI)**	**Corrected p-value**

**Time required**										
no	315	69.1	66	21.3	244	78.7	χ^2 ^= 20.13	< 10-3	2.55	< 10-3
yes	139	30.8	57	41.9	79	58.1	1df		(1.63-3.99)	
**Pressure exerted**										
no	429	95.1	109	25.7	315	74.3	χ^2 ^= 15.06	< 10-3	5.19	< 10-3
yes	22	4.9	14	63.6	8	36.4	1df		(2.09-12.91)	

Odds ratio and p-value were adjusted for age, sex and educational level.

## Discussion

In this exploratory study, we focused on online game habits and problematic overuse in adult MMORPG gamers, comparing three different instruments that could help to screen subjects with MMORPG problematic overuse. Concerning IA scales, we observed that the positivity rate observed with GIAD was higher than that observed with ISS and this confirmed that these 2 scales screened different dimensions (GIAD estimated dependence and addiction whereas ISS estimated addiction only). The superior rates obtained with GIAD mean that in substance use disorders, dependence is more frequent than addiction [37]. Moreover, for the 3 instruments used, the trend was the same but no complete concordance was observed. Also, these 3 tools did not estimate the same entities, suggesting a difference between IA and online gaming addiction. This strengthens our working hypothesis of the need for specific tools for the Internet and other specific tools for MMORPG. We showed that the adapted substance DSM-IV-TR scale (named DAS) could be a good first-line instrument to evaluate MMORPG overuse.

While the literature has documented an increasing interest in MMORPG, no consensus currently exists concerning a validated scale for determining MMORPG addiction specifically. Most previous studies look at a particular adolescent population in relation to the Internet generally, and rarely focus on video games [38]. The psychometric properties of IA scales are promising [15,39,40], whereas others have based their research on gamer interviews [14,17,38]. In addition, previous studies do not differentiate between the Internet and online video games, nor between different types of online video games [41]. MMORPGs were more likely to be associated with problematic use [33] than non-MMORPG games because MMORPG gamers tend to spend much more time playing [13].

Our study has a number of limitations. Firstly, the representativeness of the sample analyzed here could be problematic. Participants were not randomly chosen, and participation was voluntary (subjects accepted to take part in the assessment on reaching the webpage for the online questionnaire). Probably not all types of MMORPG gamers were included in this study, especially hardcore (because the responses would cause them to waste time that could be spent playing) or casual gamers (because they may feel unconcerned by the study). On the other hand, online gamers are by definition difficult to reach in any other way apart from the internet. Secondly, we focused on a specific sample (French adult MMORPG gamers only). Our results are nevertheless comparable to American and Asian studies in terms of age, gender, and family and marital status [14,33,34]. Additionally, the average time spent gaming observed here was similar to other studies [33]. Thirdly, the assessments were only based on self-reports. Responders may have been defensive in their answers, *i.e*. attempting to appear socially normal, which is an inevitable risk with any research based on self-reporting. Nevertheless, the guarantee of data anonymity may have encouraged gamers to provide honest answers. Fourthly, it was unlikely that the same gamer would respond to the questionnaire more than once because of its length (45 min). Moreover, as explained in the Results section, quality control of data eliminated inconsistent questionnaires. Fifthly, the concordance of the self-reported gradation of gaming engagement (Casual, Hardcore gamer and No life) and DAS positivity, and the different adverse effects reported suggested honest responses from a community which was cautious about providing information which may harm the public image of online games.

In terms of baseline characteristics, our study showed that French adult MMORPG gamers are often young, employed, adult University graduates, and tend to live alone in urban areas. Interpersonal interactions (77.5%) were the main attraction of this MMORPG according to their self-assessment, and not the role-play *per se *(30.9%). A young age of online gaming onset was a stronger variable associated with DAS positivity compared to the number of years of play. We observed the same number of years playing online video games for both groups [8.54 years (Standard Deviation (SD): 6.66; 95%CI: 7.81-9.26 for the DAS^- ^group *versus *8.41 years (SD: 5.93; 95%CI: 7.35-9.46) for the DAS^+^] (data not shown).

We chose to adapt the DSM-IV-TR substance dependence scale for online video games because excessive involvement in online games can be described as a form of behavioral addiction in which behavior is defined by gaming activity. This position was reinforced by the A.P.A.'s recent discussion and stance on the issue [7,21,42], which took place as we were preparing this manuscript. In addition, the criteria for problematic video game playing including tolerance, derived from the diagnostic criteria of substance dependence [25]. Furthermore, the adapted DSM-IV pathological gambling scale raised validity issues due to various distinct qualitative differences between gambling and gaming [23]. Here, we observed that for the 3 scales, the addiction rate was higher compared to other studies [14,17]. Addiction screening tools used in our study showed high and significant (p < 10^-3^) concordance when screening positive or negative: DAS and ISS showed 77.5% of concordant pairs, and DAS and GIAD showed 72.5% of concordant pairs. The higher rates for IA in our study (32.5% of ISS^+ ^and 44.3% of GIAD^+^) compared to the literature could be explained by the sample characteristics: online gamers are more likely to overuse Internet vector for gaming as well as for other Internet activities. Moreover, these two tools did not measure the same dimensions. ISS focused on addiction with loss of control and the persistence of the behavior despite adverse consequences in important areas; whereas GIAD evaluated tolerance and withdrawal symptoms in relation to dependence.

The difference in the positivity level observed for the 3 instruments underlined a real difference between gaming and generalized PIU, and requires specific tools for each field of IA. We showed that, of these three different instruments, the DAS seemed to be a valuable screening instrument for MMORPG addiction. The DAS appeared to be the one most associated with the other entities studied. This was explained firstly by the fact that ISS and GIAD scales were dedicated to the Internet, so they included other elements beside gaming, whereas DAS was specific to online video games. Secondly, we observed several analogies between DAS positivity and other addictions for which the usual scales were validated, such as alcohol addiction. For example, the odds ratio associated with a positive response to the question "Increasing time spent on the Internet to obtain satisfaction" was high (OR: 2.99); this effect could be defined as a tolerance phenomenon, which is classically found in substance addiction [28]. It is likewise well established that addiction to substances such as alcohol is associated with health and social difficulties such as family and work problems [43]. Examining behavior related to MMORPG addiction allowed us to define a "gaming adult population at risk of addiction" with numerous implications for health and personal behavior, as observed during the preparation of this manuscript by Billieux *et coll*. [44]. Indeed, gamers from all positive groups were younger than those in negative groups and were less likely to be University graduates (48.2% had at least a High School Diploma) compared to the general population of our study, which is consistent with the fact that younger gamers considered themselves more addicted [14,45]. Because of similarities with previous studies [14,15,17,33,34], a multivariate logistic regression analysis was carried out with adjustments for age, sex and educational level. All variables studied here (25/25) remained significant in the final model after adjustment. In terms of gamer characteristics, positive group gamers spent more time on the Internet per week than negative group ones and more time gaming than the population as a whole. Additionally, there was a strong relationship between the definition given by participants (Casual, Hardcore gamer or No life) and addiction level: the higher up the scale definition is, the more dependent the gamer is compared to the overall population. Gamers who felt greater personal satisfaction, sense of power or of belonging to a group and did not sleep restfully were more often in the DAS^+ ^group. DAS^+ ^group gamers also slept fewer hours per night than DAS^- ^ones and suffered sleep deprivation or diurnal sleepiness. As in Hussain's study [46], gamers claiming to feel more irritable and more anxious were more addicted than those who said they felt happier. Seeking and obtaining pleasure from games could be a protective factor from excessive gaming. Unsurprisingly, gamers claiming to be sadder were also 12 times more likely to be associated with the DAS^+ ^group than those who said they were happier. This could be due to a mood improvement sought in the game, or a consequence of adverse effects related to excessive gaming. In terms of health, players with self-reported physical or psychological effects linked to gaming were also more often in the DAS^+ ^group, and this association was 14 times more likely to be found when gamers reported both kind of adverse effects. We observed the same relationship between DAS positivity and confusing real life with fiction. In the same way, gamers who felt that guilds required time and exerted pressure were more often in the DAS^+ ^group. These feelings could be explained by the need to belong to a guild to progress in the game, to reach high levels. Guilds often organize raids and other events requiring planning, which could create a sense of obligation for members [47]. Some guilds select members who are most available and have been gaming the longest, with the aim of competing with other guilds. Moreover, we observed that guilds protected gamers, as the risk of DAS positivity increased in gamers who felt that guilds made demands on their time compared to gamers who did not feel this. Finally, as far as social impairments are concerned, DAS^+ ^group gamers appeared to go out less, see fewer friends, have marital, family, work and financial difficulties and deprive themselves of necessary purchases to play, as observed in other addictions.

In view of these results, this study underlines the fact that DAS seemed a good first-line instrument for screening gamers who could be at risk of online excessive gaming. Gamers with some of the characteristics mentioned above were not necessarily addicts but appeared to be at a substantial risk of addiction. From a public health point of view, it was therefore important to identify this population in order to describe the phenomenon in sufficient detail.

## Conclusions

This prospective study provided socio-demographic data in a large sample of adult online recruited MMORPG gamers, and tested an instrument for determining the risk factors for video game addiction. Our results confirm the need to establish health prevention programs such as Internet-based prevention for MMORPG abuse and a Centre for Online Addiction, as initiated by Young, including online consultations (http://www.netaddiction.com) and treatment such as cognitive behavioral therapy [48].

## List of abbreviations

A.P.A.: American Psychiatric Association; CI: Confidence Interval;DAS: DSM-IV-TR substance dependence scale adapted for MMORPG; DSM-IV-TR: Diagnostic and Statistical Manual of Mental Disorders, Fourth Edition-Text Revision; GIAD: Goldberg Internet Addiction Disorder scale; IA: Internet addiction; IAT: Internet Addiction Test; IP: Internet Protocol; ISS: Internet Stress Scale of Orman; MMORPG: Massively Multiplayer Online Role-Playing Game; PIU: Problematic Internet Use; PVP: Problem Videogame Playing; OR: Odds Ratio; SD: Standard Deviation; WHO: World Health Organization; WoW: World of Warcraft^®^

## Competing interests

The authors declare that they have no competing interests.

## Authors' contributions

SA, FM and EH planned, designed the study and wrote the protocol. SA, MN and BT undertook the acquisition of the data. JM, PV and DS researched the literature. MN, FM, EH, PG managed analyses and interpretation of the data. FM undertook the statistical analyses. SA and MN wrote the first draft of the manuscript. EH and PG supervised the study. All authors contributed to the critical revision of the manuscript and have approved the final version.

## Pre-publication history

The pre-publication history for this paper can be accessed here:

http://www.biomedcentral.com/1471-244X/11/144/prepub
